# Concise Review: Vascular Stem Cells and Tumor Angiogenesis

**DOI:** 10.1002/stem.583

**Published:** 2010-12-23

**Authors:** Juan M Melero-Martin, Andrew C Dudley

**Affiliations:** 1 Department of Cardiac Surgery, Children's Hospital BostonBoston, Massachusetts; 2 Department of Surgery, Harvard Medical SchoolBoston, Massachusetts; 3 Vascular Biology Program, Children's Hospital BostonBoston, Massachusetts

**Keywords:** Tumor, Angiogenesis, Stem cells, Adult stem cells, Mesenchymal stem cells, Bone marrow, Tumor stroma, Hematopoietic stem cells, Endothelial progenitor cells, Tumor microenvironment

## Abstract

Solid tumors are complex “organs” of cancer cells and a heterogeneous population of hematopoietic cells, mesenchymal cells, and endothelial cells. The cancer stem cell model proposes that tumor growth and progression is driven by rare populations of cancer stem cells; however, nontumor-forming stem and progenitor cells are also present within the tumor microenvironment. These adult stem cells do not form tumors when injected into experimental animals, but they may augment tumor growth through juxtacrine and paracrine regulation of tumor cells and by contributing to neovascularization. Thus, cancer cells may actively co-opt nontumor-forming stem cells distally from the bone marrow or proximally from nearby tissue and subvert their abilities to differentiate and maintain tissue growth, repair, and angiogenesis. This review will cover the roles of nontumor-forming vascular stem cells in tumor growth and angiogenesis. Stem Cells 2011;29:163–168

## CANCER STEM CELLS

The suggestion that tumors may be characterized by hierarchies of organized stem cells was a paradigm shift in our understanding of tumor biology [1]. Putative cancer stem cells (CSCs) have now been identified in several tumor types, and several competing but not necessarily mutually exclusive models have been put forth to explain tumor cell heterogeneity and propagation of CSCs. A stochastic model states that all cells within a tumor have equal abilities to initiate and maintain tumor growth, whereas a hierarchical model suggests that only a minor subset of cancer cells can self-renew and give rise to tumor-forming and nontumor-forming progeny. However, the rarity of stem cells in tumors has been brought into question, as has their existence at all in some mouse tumor models (reviewed in [2]). Normal stem cells and CSCs can be identified by efflux of Hoechst dye via multi-drug resistance (MDR) and ATP-binding cassette transporters [3]. But the ability to export Hoechst dye diminishes in more committed progeny. By inference, refractoriness to chemotherapy could be explained by rapid export of cytotoxic agents by the CSC pool. Thus, CSC may act as the “linchpin” in some solid tumors and their selective elimination may initiate a cascade ultimately leading to tumor destruction. Notably, cancer cells with stem-like properties that express markers of vascular endothelium and form tumor blood vessels have been described [4]. However, second populations of “adult” stem/progenitor cells, which do not form tumors, are also present in the tumor microenvironment and are the building blocks of most tumor blood vessels. This review covers the role of this second population of nontumor-forming progenitors and their role during angiogenesis and tumor progression.

## DEFINITION OF STEM AND PROGENITOR CELLS

First, a distinction should be made between bona fide stem cells that have the capacity for unlimited self-renewal and multilineage differentiation (e.g., embryonic stem cells) and progenitor cells or “precursor cells” that have limited self-renewal and differentiation abilities. The generation of progenitor cells is hierarchical with an undifferentiated parental cell lying at the apex that generates progressively more differentiated progeny. For example, hematopoietic stem cells, which reside in the bone marrow, generate an array of more committed hematopoietic progenitor cells (HPC) that act as specific precursors to different blood cell lineages. Mesenchymal stem cells (herein termed mesenchymal progenitor cells [MPCs]) although often referred to as stem cells, are also a type of progenitor or “precursor” cell. MPCs are found throughout the body, they can differentiate to form multiple cell types (e.g., osteocytes, adipocytes, chondrocytes, fibroblasts, and smooth muscle cells) and they have a limited ability to self-renew. Endothelial progenitor cells (EPCs) are another type of lineage-restricted progenitor cell. Although there is still debate about the definitive site(s) where EPC reside in the body, the progeny of circulating EPC (defined as endothelial colony-forming cells [ECFCs]) circulate in peripheral blood, can be ex vivo expanded into clonal populations and will form vessel lumens when injected into mice. EPCs, MPCs, and HPCs are often identified in blood or tissues using the markers listed in Table 1.

**Table 1:** Common markers used to identify EPC, MPC, HSC, and HPC

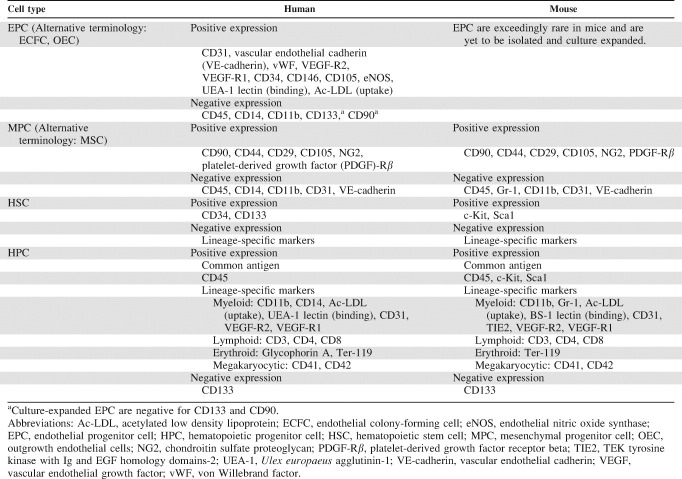

## ENDOTHELIAL PROGENITOR CELLS

EPCs may be defined as circulating precursor cells with the ability to differentiate to form functional blood vessels at sites of neovascularization. However, there has been a lack of consensus regarding EPC definition, origin, and function in humans and animal models of disease. As a consequence, the contribution of these cells to the vasculature of solid tumors is still the subject of lively debate [5].

A seminal article identified EPC as cells that circulate in peripheral blood and express markers such as CD34 and vascular endothelial growth factor receptor-2 (VEGF-R2) (kinase insert domain receptor [KDR]) [6]. However, hematopoietic cells also express these cellular markers, and they can be mobilized into circulation from the bone marrow in order to home to sites of neovascularization. The functional differences distinguishing hematopoietic cells (generally do not line blood vessels) from bona fide ECFC (form lumens) are becoming clear. Yoder et al. suggest that most of the cells long referred to as EPC are in fact hematopoietic cells that are proangiogenic and assist during nascent vessel formation but have no ability to form vessel lumens (reviewed in [7]).

The discrepancies surrounding the definition of EPC have made it difficult to identify these elusive cells in vivo, especially in tumors. Bone marrow has been proposed as a depot for circulating EPC in humans and in mouse tumor models although it is not always clear whether the identified cells are ECFC or hematopoietic cells located close to the blood vessel wall (reviewed in [8]). The absolute number of putative bone marrow EPC is suggested to differ dramatically depending on the tumor site, mouse strain, stage of tumor progression, and method of quantification (reviewed in [9]). Gao et al. [10] suggest that EPC facilitate metastasis as their genetic ablation using ID1 shRNA mitigated pulmonary micro metastasis and impaired angiogenesis. Some chemotherapies are reported to increase EPC in circulation and their homing to tumors following vascular damage [11]. On the other hand, some studies report minimal contribution of EPC to the tumor vasculature and question the importance of these cells during tumor growth [12] (for further discussion see [13]). Our lab recently showed that the endothelium in spontaneous prostate tumors was rarely, if ever, derived from the bone marrow and instead most likely recruited from nearby tissue [14]. Interestingly, we also found no difference in the number of incorporated circulating endothelial cells in prostate tumors versus normal age-matched prostates. Studies in humans using sex-mismatched bone marrow transplants also suggest a minor but steady-state incorporation of donor-derived endothelium in normal tissues at levels similar to those reported in human cancers [15].

Recently, the vascular wall itself has been proposed as a source for EPC because it contains subpopulations of endothelial cells with properties similar to blood-derived ECFC [16]. Because endothelial turnover in resting blood vessels is low, a local and immediate reservoir of highly proliferative endothelium proximal to the site of injury may be biologically advantageous following tissue injury. Vessel wall EPC (VW-EPC) are proposed to rest in a “vasculogenic zone” between the smooth muscle and advential layers at the periphery of large blood vessels (reviewed in [17]). No studies to date have determined whether VW-EPC might form the majority of the angiogenic response in tumors, if there are any unique properties in VW-EPC that could be exploited as an antiangiogenesis strategy or if VW-EPC might mediate vascular rebound following treatment with antiangiogenic therapies. Definitive proof for the existence of VW-EPC will have to wait on the identification of a unique marker that can distinguish these cells from mature vascular endothelial cells.

## MESENCHYMAL PROGENITOR CELLS

MPC and their progeny are present in most if not all postnatal organs. Their major function is to maintain and regenerate connective tissues and to replenish damaged tissue following injury or inflammation. MPC differentiation is dependent on the local production of cytokines/growth factors resulting in tissue specificity. Therefore, MPC may differentiate into the type of tissue they engraft [18]. Because paracrine/juxtacrine interactions between MPC and neighboring cells may be critical for controlling the rate and timing of differentiation, it is predicted that crosstalk between mutant cancer cells and resident MPC could affect their normal differentiation patterns. A good example is the adipogenic differentiation of MPC in subcutaneous tumor sites and osteoblastic differentiation at sites of lung metastasis [19]. Similar to HPC, MPC may be hierarchically organized into heterogeneous clonal variants with different proliferative and differentiation abilities. Although it has not been investigated, these variants may function differently depending on tumor stage or type.

MPC may form a large proportion of the reactive stroma in solid tumors and share properties with carcinoma-activated fibroblasts, which express alpha smooth muscle actin (α-SMA), however, it is difficult to distinguish bona fide MPC from fibroblasts due to overlapping expression of several surface markers. Recently, Hoechst dye efflux was used to isolate and culture expand MPC from prostate carcinoma [20]. One unanswered question is whether bone marrow is the primary source of MPC in tumors or if there are alternative sites. Factors that typically stimulate angiogenesis including hypoxia, ischemia, and proinflammatory cytokines also stimulate MPC mobilization; thus, intravenously delivered MPC display tropism to sites of injury and engrafted tumors [21]. Notably, marrow-derived PDGF-Rβ^+^ progenitor cells were shown to localize in a perivascular position raising the possibility that circulating MPC or MPC-like cells “seek out” blood vessels in the tissue they engraft [22]. On the other hand, tumor-associated MPC may be co-opted from nearby tissue (e.g., adipose tissue) where they reside in a perivascular niche. For example, Crisan [23] showed that pericytes and MPC are indistinguishable sharing common features such as multipotency. Thus, MPC in tumors are conveniently positioned to form pericytes, which are major structural and paracrine cellular components of new blood vessels.

The effect of MPC on primary tumor growth is equivocal with studies showing enhancement [24], inhibition [25], or no effect [24] when coinjected with different tumor cell lines. A recent study showed that neutralization of LL-37, a proinflammatory peptide, reduced engraftment of MPC in human ovarian xenografts resulting in tumor growth inhibition [26]. On the other hand, due to their tumor-homing abilities, MPC may be exploited as vectors for drug delivery. Studeny et al. [27] loaded MPC with interferon beta (IFN-β), injected them intravenously, and found suppression of tumor growth and metastasis and prolonged survival in mice. Similar to their effect on primary tumor growth, the role of MPC in metastasis is conflicting. Karnoub [24] reported that MPC increased metastasis when admixed with various tumor cell lines. Other studies show that intravenous (i.v.) injection of MPC has either no effect on 4T1 breast cancer metastasis [19] or reduces metastasis when MPC are transduced with *TRAIL* [28]. Some of these discrepancies are likely due to the different routes of MPC injection (coinjection with tumor cells vs. i.v. injection).

The tumor-promoting properties of MPC might be related to angiogenesis stimulation. MPC differentiate to form pericytes and are a major source of VEGF secretion [29]. Although controversial, transdifferentiation of MPC to directly form endothelium has been reported [30]. Similar to their role played during wound healing, tumor-associated MPC may secrete proinflammatory chemokines, which recruit proangiogenic hematopoietic cells (e.g., monocytes and neutrophils) [31]. On the other hand, one study suggested that MPC dose-dependently inhibited angiogenesis due to reactive oxygen species generation, which led to endothelial apoptosis and vessel regression [32]. Together, MPC and their progeny may have both protumor and antitumor properties that are tumor-type dependent or related to the different routes of MPC injection. Furthermore, MPC might be exploited as drug delivery vehicles due to their tumor homing properties. Although laboratory mice are good models for studying the role of MPC in tumors, their function in clinical cancers has not been investigated in detail.

## HPCs AND THEIR PROGENY

The role of hematopoietic cells in tumor angiogenesis has recently received great attention. HPC from the bone marrow emigrate to the blood and tissues and differentiate to form cells of the innate and adaptive immune system. Tumor-infiltrating leukocytes can either protect against or promote tumor formation. The simplest example of the former is loss of immune surveillance and spontaneous tumor formation in some strains of immunodeficient mice. On the other hand, CD4^+^ T lymphocytes may promote tumor cell invasion and metastasis by altering the function of tumor-associated macrophages (TAMs) [33]. Myeloid lineage cells, including monocytes/macrophages, neutrophils, dendritic cells, eosinophils, and mast cells feature prominently in the stroma of most solid tumors (reviewed in [34]). Although direct stimulation of malignant cells by these hematopoietic cells is possible, the tumor-promoting abilities of the recruited myelomonocytic pool is largely related to their secretion of a variety of proangiogenic factors and ability to orchestrate the formation of new blood vessels.

One of the first clues that HPC might be required for angiogenesis came from studies in the developing embryo. For example, acute myeloid leukemia 1 (AML1)–deficient embryos, which lack definitive hematopoiesis, showed impaired angiogenesis in the head and pericardium that was rescued by addition of HPC-expressing angiopoietin 1 (ANG-1) [35]. Thus, even physiological angiogenesis is dependent on crosstalk between the remodeling endothelium and recruited HPC. Moreover, we showed that “engineered” blood vessels in the adult mouse are also dependent on hematopoietic cell recruitment, as selective elimination of GR-1^+^-circulating myeloid cells impaired blood vessel development and anastomosis with host vasculature [36]. Pathological angiogenesis (e.g., solid tumors) is also characterized by recruitment of an array of myelomonocytic cells with overlapping phenotypes and functions (reviewed in [37]). For example, a novel population of proangiogenic TEK tyrosine kinase with Ig and EGF homology domains-2 (TIE-2)–expressing monocytes was recently identified in tumors [38]. Selective elimination of TIE-2 monocytes impairs tumor growth and angiogenesis. On the other hand, similar to MPC, TIE-2 monocytes (and other HPC) may be used as vectors for drug delivery due to their tumor-homing properties.

The catalytic function of hematopoietic cells during tumor progression may be related to their expression of proangiogenic and tissue-remodeling factors including MMPs and VEGF. Furthermore, tumor-derived factors may activate or change the phenotype of the recruited pool of hematopoietic cells. A good example is the M2 skewing of TAMs as tumors become more vascular and invasive [39]. Macrophages invade most solid tumors in great numbers and elicit inflammatory responses that are generally protumorigenic and proangiogenic. The numbers of TAMs in Hodgkin's lymphoma is associated with a poor patient prognosis although the factor(s) mediating their tropism to the tumor site were not identified [40]. A hypoxia inducible factor alpha (HIF1α)/chemokine ligand 12 (CXCL-12) axis was shown to mobilize proangiogenic MMP9-expressing monocytic cells from bone marrow, which controls a postulated angiogenic switch in tumors [41]. These marrow-derived monocytic cells are important mediators of resistance to anti-VEGF therapies [42] and angiogenesis rebound following radiotherapy [43]. Thus, depletion of specific populations of HPC and their progeny can have a favorable antitumor response, perhaps by indirectly controlling the rate of angiogenesis.

In addition to their paracrine functions, myeloid-lineage cells may act as “vascular bridges” by guiding and connecting nascent blood vessels. Proangiogenic TIE-2^+^ macrophages, for example, were recently shown to guide tip-cell fusion, a critical step during blood vessel anastomosis [44]. The ability of myeloid-lineage cells to directly form blood vessels is also possible. Transdifferentiation of CD45^+^ myeloid cells to form endothelial-like cells was described by Bailey [45]. Yang et al. [46] found that Gr-1^+^/CD11b^+^ cells acquired properties of endothelial cells in tumors, expressed VE-cadherin and lined blood vessels. Similarly, lymphatic vessel endothelial hyaluronan receptor-1 (LYVE-1)^+^ macrophages were shown to differentiate to form lymphatic endothelium [47], a feature probably related to their inherent plasticity. Taken together, myeloid-lineage cells, through diverse mechanisms, may facilitate angiogenesis and enable tumor progression. It will be important to further clarify the factors mediating trafficking and differentiation of myeloid cells at the tumor site. The use of “humanized” mice engrafted with tumor cells could be an important tool for dissecting the function of HPC and their progeny in the laboratory.

## PERSPECTIVE: Do TUMORS SUBVERT PROGENITOR CELL FUNCTIONS?

**Figure 1:**
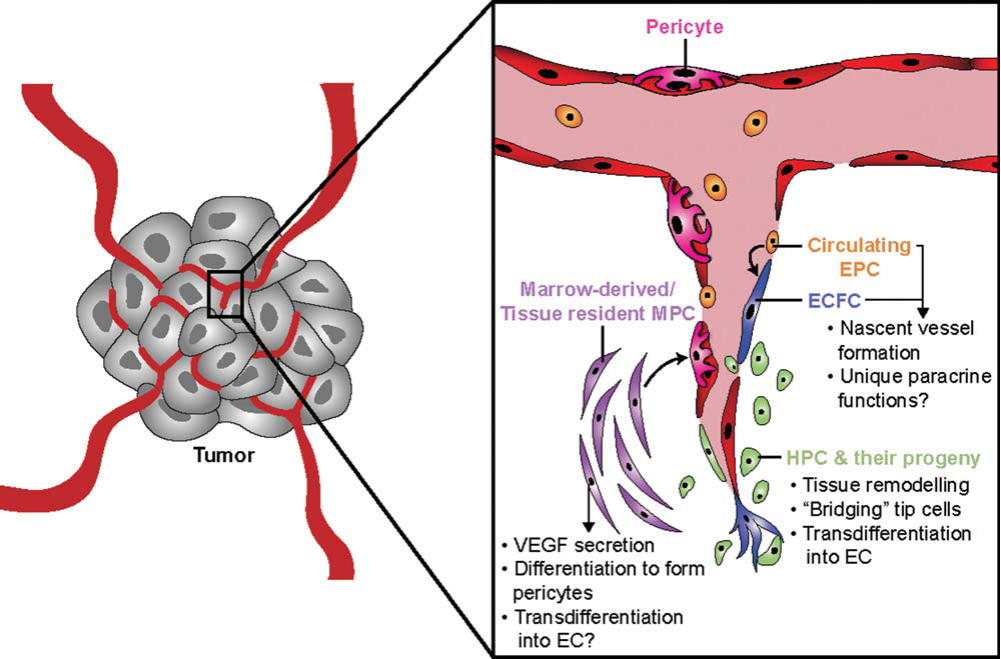
Functions of vascular stem cells in solid tumors. MPC derived from the bone marrow or nearby tissues are a source of VEGF, a potent endothelial cell survival and motility factor. MPC may also differentiate to form pericytes that provide structural support to the nascent vasculature. HPC and their progeny act as “accessory” cells during angiogenesis by expressing tissue remodeling and endothelial survival factors such as MMP9 and VEGF. Similar to MPC, they may provide paracrine instructions to sprouting tip cells during anastomosis. EPC may give rise to ECFC and constitute the primary lumen-forming cells of angiogenic sprouts. ECFC may also provide unique paracrine cues to neighboring “adult” endothelium or the recruited pool of HPC. Taken together, these three populations of progenitors work in concert to form the building blocks of tumor blood vessels. ECFC and MPC form lumens and perivascular cells, respectively, whereas HPC direct and orchestrate vessel sprouting and anastomosis. Abbreviations: EC, endothelial cell; ECFC, endothelial colony-forming cell; EPC, endothelial progenitor cell; HPC, hematopoietic progenitor cell; MPC, mesenchymal progenitor cell; VEGF, vascular endothelial growth factor.

Solid tumors are caricatures of a dysfunctional “organ.” In the shadow of malignant cells is the resident population of mesenchymal, hematopoietic, and endothelial cells that constitutes the tumor stroma. More than innocent bystanders, these stromal cells provide a growth advantage, for example, by forming the building blocks of tumor blood vessels (Fig. 1). Prolonged paracrine/juxtacrine interactions between malignant cells and stromal cells might result in unexpected patterns of differentiation and plasticity, particularly in the resident population of more primitive adult stem/progenitor cells. These subtle changes in the stroma may accelerate or even precede progression to malignancy.

A clue that malignant cells might impinge on the differentiation state in the stroma comes from studies identifying aberrant DNA methylation and chromatin modification patterns in cancer-associated fibroblasts. Multiple tumor types, for example, breast and prostate are characterized by stromal alterations in methylated DNA (reviewed in [48]). Because normal patterns of differentiation are controlled, in part, by epigenetic switches, a “rogue” differentiation state of tumor-associated stromal cells may emerge. For example, we have shown that prostate tumor endothelial cells have unusual properties including acquisition of stem-like features and bipotent differentiation to form bone and cartilage [49]. A second level of control of stem cell fate and plasticity can occur through mechanical cues relayed by interactions between stem cells with the extracellular matrix ECM; reviewed in [50]). ECM remodeling and stiffening is common in the stroma of most tumors. For example, the breast tumor microenvironment is characterized by highly cross-linked collagen that modulates focal adhesions and alters cellular signaling [51]. It will be interesting to determine how changes in the tumor microenvironment affect the timing and pattern of differentiation in resident progenitor cells. Furthermore, whether tumors can “reprogram” the resident pool of progenitor cells, hijacking their normal functions, to facilitate tumor progression and metastasis should be investigated.

Pathologists have long noted the heterogeneous and disorganized nature of parenchymal cells in tumors. As recently suggested, these stromal cells may coevolve with tumor cells during tumor progression and acquire characteristics of tumor cells nearby [48]. “Normalizing” the tumor microenvironment to reprogram the fate and plasticity of progenitors within the stroma, in addition to direct targeting of tumor cells, might be an effective strategy for reversing or eliminating some cancers.

## CONCLUSION

Solid tumors may be characterized by two populations of stem/progenitor cells: CSC that may ultimately drive tumor growth and progression and nontumor/forming progenitors, some of which comprise the building blocks of tumor blood vessels. Malignant cells and their supporting stromal cells are held together by an extracellular matrix. Tumors may thus resemble dysfunctional organs able to recapitulate features of development while malignant cells provide corrupt instructions to surrounding tissues [52]. Understanding how tumors alter their own microenvironment, in ways that promote their progression and perhaps ability to metastasize, is critical for the design of therapies that might eliminate some cancers.
